# Tyrosine kinase inhibitor and rituximab‐CHOP treatment for concurrent chronic myeloid leukemia and non‐Hodgkin lymphoma: a case report

**DOI:** 10.1002/ccr3.1253

**Published:** 2017-11-02

**Authors:** Yuki Takeyasu, Atsushi Satake, Yoshiko Azuma, Yukie Tsubokura, Hideaki Yoshimura, Masaaki Hotta, Takahisa Nakanishi, Shinya Fujita, Aya Nakaya, Tomoki Ito, Shosaku Nomura

**Affiliations:** ^1^ First Department of Internal Medicine Kansai Medical University Osaka 573‐1010 Japan

**Keywords:** Chronic myeloid leukemia, myelosuppression, non‐Hodgkin lymphoma, primary mediastinal large B‐cell lymphoma, tyrosine kinase inhibitor

## Abstract

Non‐Hodgkin lymphoma can occur concurrently with chronic phase‐chronic myeloid leukemia (CML) at initial diagnosis. Combination treatment with second‐generation tyrosine kinase inhibitors and rituximab‐CHOP for patients newly diagnosed with CML and non‐Hodgkin lymphoma is effective for both diseases. However, we found that this treatment combination may induce severe myelosuppression.

## Introduction

Chronic myeloid leukemia (CML) is a clonal proliferative disorder of hematopoietic stem cells characterized by the Philadelphia (Ph) chromosome created by a reciprocal t(9:22) translocation, which transfers the Abelson (*ABL*) oncogene on chromosome 9 to the breakpoint cluster region (*BCR*) of chromosome 22, thus resulting in a fused *BCR/ABL* gene. The prognosis of CML has dramatically improved as the development of BCR‐ABL tyrosine kinase inhibitors (TKIs) 1. Although TKIs are unlikely to induce secondary malignancies 2, CML patients sometimes develop other malignancies because of their prolonged survival. However, coexistence of CML and another hematological malignancy at diagnosis is rarely observed. Moreover, the optimal treatment strategy for these patients in the second‐generation TKI (2nd TKI) era remains poorly understood. We describe a case of concurrent CML and NHL treated with 2nd TKI+rituximab‐CHOP (R‐CHOP) therapy.

## Case Report

**Figure 1:**
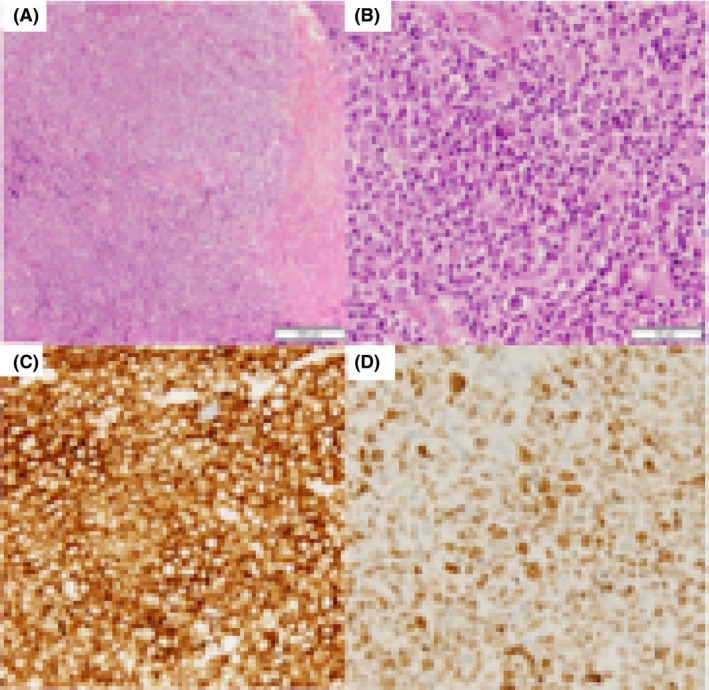
Histopathological images. Hematoxylin and eosin staining of the mediastinal tumor biopsy specimen (A, ×100) (B, ×400) revealed focal and colonized proliferation of large lymphoid cells. Immunohistochemical stains highlight that large lymphocytes are positive for CD 20 (C, ×400) and bcl‐6 (D, ×400). CD, cluster of differentiation; bcl‐6, B‐cell lymphoma 6.

**Figure 2:**
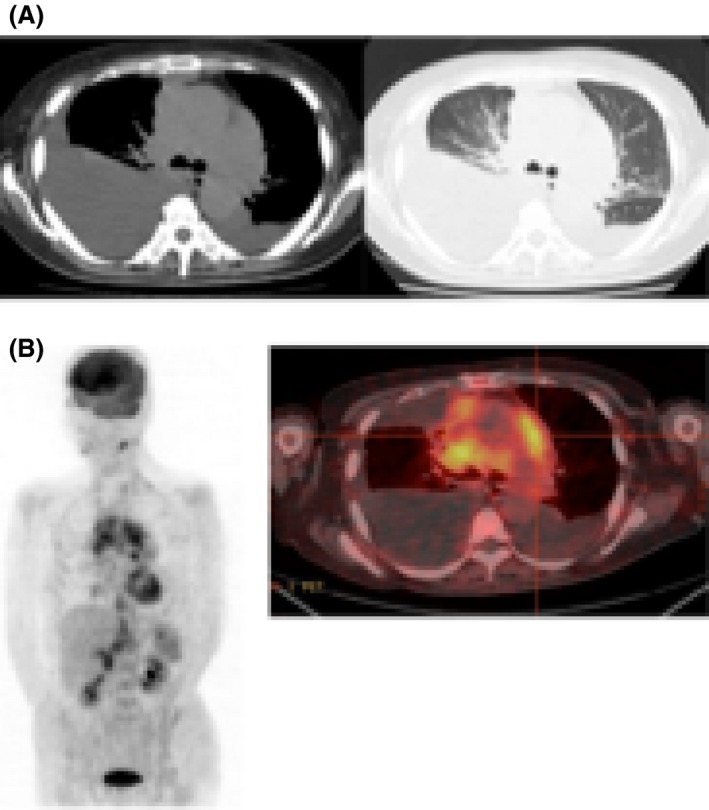
Imaging findings. (A) Computed tomography images at initial consultation. (B) FDG positron‐emission tomography images obtained before R‐CHOP. The image shows FDG accumulation in the cervical, mediastinal, hilar, and abdominal lymph nodes. FDG, 18F‐Fluorodeoxyglucose; R‐CHOP, Rituximab‐CHOP.

A 66‐year‐old woman diagnosed with leukocytosis and a mediastinal tumor was referred to our hospital for further investigation. Physical examination revealed significant splenomegaly (10 cm below the costal margin), but no enlarged superficial lymph nodes. Laboratory test findings were as follows: white blood cell count, 281.9 × 10^9^/L (29% neutrophils, 0% lymphocytes, 1.5% monocytes, 7.0% basophiles, 4.5% myeloblasts, 35.0% myelocytes, and 9.5% metamyelocytes); hemoglobin, 9.5 g/dL; platelet count, 41.9 × 10^4^/*μ*L; lactic dehydrogenase, 833 U/L; and vitamin B12, >1500 pg/mL. Major BCR‐ABL mRNA transcripts were detected from peripheral blood via polymerase chain reaction. Bone marrow analysis showed marked hypercellularity with significant myeloid hyperplasia, while G‐band chromosome analysis showed a Ph‐chromosome translocation t(9;22) (q34;q11.2) in 100% of metaphases (20/20 cells) analyzed. The Sokal score was 1.686, indicating high risk. Computed tomography showed a tumor extending from the mediastinum to the right lung hilar region and bilateral pleural effusion. Therefore, she was diagnosed with CML; the mediastinal tumor was considered an extramedullary CML lesion. Dasatinib, which is applicable for chronic‐phase, accelerated‐phase, and blastic‐phase CML, was administered at a dose of 100 mg/day. Flow cytometric analysis of the pleural effusion did not indicate monoclonal abnormal leukocytes, while cytogenetic analysis of the fluid showed the Ph‐chromosome in 15% of metaphases (3/20 cells). Hematologic response was attained immediately; however, dyspnea owing to the increased pleural effusion emerged 5 weeks after the administration of dasatinib. Therefore, dasatinib was discontinued, and a diuretic drug was administered. Nevertheless, the pleural effusion worsened 2 weeks later. Dasatinib treatment was restarted at a dose of 50 mg/day. Subsequently, the patient underwent mediastinal tumor biopsy via video‐assisted thoracoscopic surgery. Histopathological examination showed focal and colonized proliferation of large lymphoid cells (Fig. 1A and B). Immunohistochemical analysis indicated these lymphocytes were CD3‐negative, CD20‐positive, and bcl‐6‐positive (Fig. 1C and D). Fluorescence in‐situ hybridization of the biopsy sample demonstrated the absence of the *BCR/ABL* fusion gene. 18F‐Fluorodeoxyglucose‐positron‐emission tomography/computed tomography demonstrated fluorodeoxyglucose accumulation (SUVmax 5.9) with lymphadenopathy in the cervical, mediastinal, hilar, and abdominal lymph nodes (Fig. 2). Finally, the patient was diagnosed with concurrent chronic‐phase CML (CP‐CML) and primary mediastinal large B‐cell lymphoma (PMBL).

**Figure 3:**
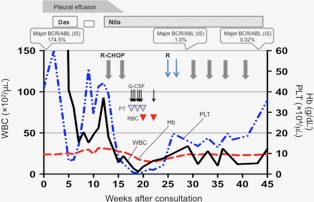
Clinical course from the initial consult in our hospital. Das, dasatinib; Nilo, nilotinib; R, rituximab; PT, platelet transfusion; RBC, red blood cell transfusion.

The patient was administered R‐CHOP therapy for the PMBL, and nilotinib (300 mg twice daily) for the CML to clear the pleural effusion. Grade 4 neutropenia occurred after the first cycle of nilotinib+R‐CHOP therapy. Furthermore, grade 4 thrombocytopenia and grade 3 anemia developed after the second cycle. Therefore, R‐CHOP therapy was discontinued owing to the prolonged severe myelosuppression. The third cycle of R‐CHOP, comprising of the same dosage as first and second cycles, was restarted 12 weeks after the previous cycle. Severe thrombocytopenia and anemia were not observed. There were no nonhematological adverse events during the treatment with nilotinib+R‐CHOP therapy. Complete remission of PMBL after six cycles of R‐CHOP was confirmed via 18F‐fluorodeoxyglucose–positron‐emission tomography/computed tomography. Disappearance of the BCR‐ABL fusion gene in peripheral blood was demonstrated via Fluorescence in‐situ hybridization analysis, 6 months after the initiation of TKI treatment, indicating a complete cytogenetic response. The BCR‐ABL mRNA transcript level in peripheral blood measured via quantitative reverse‐transcriptase polymerase chain reaction at 9 months after diagnosis revealed a major molecular response per international standards (Fig. 3).

## Discussion

In the present case, examination of the patient's bone marrow resulted in a diagnosis of CP‐CML, while the biopsy of the mediastinal tumor indicated that the PMBL originated from another clonal CML population. The patient received 2nd TKI+R‐CHOP and has achieved total remission from both diseases, despite severe myelosuppression.

Little is known about the clinical and genetic characteristics of B‐cell NHL with CML, and most of these cases have been reported before the TKI era 3, 4, 5. 2nd TKIs have shown remarkable efficacy for newly diagnosed CP‐CML 6, 7, 8, 9, 10; however, optimal approaches for patients with concurrent CML and NHL at diagnosis remains unclear.

Pleural effusions occurred more frequently in patients receiving dasatinib 1, 8, 10. Therefore, TKIs apart from dasatinib are commonly selected for patients at risk of developing pleural effusions. Until histopathological confirmatory diagnosis, we suspected that the mediastinal tumor with pleural effusion was an extramedullary lesion of CML, namely a blast crisis CML, hence, we had prescribed dasatinib treatment initially. Lymphopenia, neutropenia, and thrombocytopenia are common hematologic adverse events of nilotinib treatment in patients with newly diagnosed CP‐CML 1, 6, 7. Interestingly, these adverse events generally indicate a favorable profile. Moreover, as witnessed in our case, 2nd TKI+R‐CHOP therapy for patients with newly diagnosed CML and NHL may induce serious myelosuppression. The myelosuppression may have been caused by a small quantity of normal hematopoietic stem cells. After the achievement of a major molecular response and recovery from myelosuppression, our patient did not develop severe thrombocytopenia or anemia due to the nilotinib+R‐CHOP therapy. Therefore, if a patient with CML has achieved a good response, the efficacy of a combination of chemotherapy with another treatment may not be affected by hematologic toxicity. Secondary cancers that occur in a small percentage of patients with CML are mostly neoplasms of nonhematologic origin 2. The occurrence of NHL, mostly T‐cell lymphomas, with CML is less frequent 4. Identification of a B‐cell lymphoma at the time of CML diagnosis is even rarer, and in our case, Ph^‐^PMBL was identified in a patient with CP‐CML at diagnosis. PMBL is an aggressive lymphoma, which generally has a high SUVmax value with 18F‐Fluorodeoxyglucose–positron‐emission tomography/computed tomography; however, the SUVmax was lower than expected in our case. This might be associated with hyperglycemia as the patient had mild diabetes mellitus. There are some case reports in which patients have demonstrated concurrent CML and NHL at diagnosis 3, 5, 11. Nevertheless, to the best of our knowledge, no patient has received 2nd TKI and chemotherapy as an initial treatment. Furthermore, we believe that this is the first case of concurrent NHL and CP‐CML successfully treated with combination therapy consisting of 2nd TKI and R‐CHOP. The findings of the case also indicate that serious myelosuppression may be caused by this combination therapy early after its initiation.

In conclusion, NHL can occur concurrently with CP‐CML at initial diagnosis. Despite severe myelosuppression, treatment with 2nd TKI+R‐CHOP can be effective for patients with newly diagnosed CP‐CML and concurrent NHL. Optimal treatment strategies including the appropriate dose or timing of chemotherapy against the second malignancy should be elucidated for patients with CML receiving TKI therapy.

## Conflict of Interest

None declared.

## Authorship

YT: hospitalization of the patient and data collection. AS: hospitalization and outpatient follow‐up, data analysis, and manuscript drafting. YA, YT, HY, MH, TN, SF, AN, TI, and SN: patient's hospitalization and manuscript review. SN: supervision of the case.
